# Disruption of the monocarboxylate transporter-4-basigin interaction inhibits the hypoxic response, proliferation, and tumor progression

**DOI:** 10.1038/s41598-017-04612-w

**Published:** 2017-06-27

**Authors:** Dillon M. Voss, Raffaella Spina, David L. Carter, Kah Suan Lim, Constance J. Jeffery, Eli E. Bar

**Affiliations:** 1 Department of Neurological Surgery, Case Western Reserve University School of Medicine and The Case Comprehensive Cancer Center, Cleveland, OH USA; 2 Department of Radiation Oncology, Dana-Farber Cancer Institute, Boston, MA USA; 3 Department of Biological Sciences, The University of Illinois at Chicago, Chicago, IL USA

## Abstract

We have previously shown that glioblastoma stem cells (GSCs) are enriched in the hypoxic tumor microenvironment, and that monocarboxylate transporter-4 (MCT4) is critical for mediating GSC signaling in hypoxia. Basigin is involved in many physiological functions during early stages of development and in cancer and is required for functional plasma membrane expression of MCT4. We sought to determine if disruption of the MCT-Basigin interaction may be achieved with a small molecule. Using a cell-based drug-screening assay, we identified Acriflavine (ACF), a small molecule that inhibits the binding between Basigin and MCT4. Surface plasmon resonance and cellular thermal-shift-assays confirmed ACF binding to basigin *in vitro* and in live glioblastoma cells, respectively. ACF significantly inhibited growth and self-renewal potential of several glioblastoma neurosphere lines *in vitro*, and this activity was further augmented by hypoxia. Finally, treatment of mice bearing GSC-derived xenografts resulted in significant inhibition of tumor progression in early and late-stage disease. ACF treatment inhibited intratumoral expression of VEGF and tumor vascularization. Our work serves as a proof-of-concept as it shows, for the first time, that disruption of MCT binding to their chaperon, Basigin, may be an effective approach to target GSC and to inhibit angiogenesis and tumor progression.

## Introduction

Glioblastoma (GBM) are the most common and lethal brain tumors in adults, claiming about 14,000 lives annually in the U.S. alone^1^. Specific histological hallmarks that unify all GBM and distinguish them from lower grade gliomas include pseudo palisading cells around centers of hypoxia/necrosis and microvascular proliferation. These histological hallmarks represent specific niches within the tumor microenvironment that regulate metabolic needs, immune surveillance, and invasion. Work done by our group and others has established that stem-like GBM cells favor low oxygen levels and are typically found in the hypoxic tumor microenvironment^2–7^ (and reviewed in Bar^8^). More recently, we identified the Monocarboxylate Transporter-4 (MCT4) as a critical signaling node in GBM facilitating Hypoxia Inducible Factor -1 (HIF-1α)-dependent induction of stem-like phenotype under hypoxia^9^.

Monocarboxylate transporters (MCTs) constitute a family of proton-linked plasma membrane transporters that carry molecules having a single carboxylate group such as lactate and pyruvate across biological membranes. MCTs have been linked to the regulation of glycolytic metabolism^10^, and glycolytic metabolism is crucial for the expansion of neoplastic as well as non-neoplastic stem cells^3, 4, 8, 11–18^. The cell surface glycoprotein Basigin (also known as CD147 or EMMPRIN) has been implicated in a plethora of biological functions - including angiogenesis, proliferation, survival and invasion - and its deregulation is observed in a variety of human malignancies (reviewed in ref. 11). Indeed, MCT4 and basigin, are both commonly expressed in GBM^19, 20^ with basigin found to be a negative prognosticator for survival and crucial for MCT4 plasma membrane expression^21–23^. In a recent report, we showed that MCT4 levels are greatly induced in the stem-cell-rich hypoxic microenvironment of GBMs. Moreover, we demonstrated that targeting MCT4, using short-hairpin RNAs (shRNAs), resulted in a significant growth inhibition *in vitro* and *in vivo*. These effects were augmented by hypoxic culture conditions and did not involve perturbations in lactate homeostasis, which led us to conclude that MCT4 signaling relies on lactate export-independent mechanism for growth inhibition in GBMs^9^.

Given their crucial roles in cancer and the known dependency of MCTs on Basigin for functional plasma membrane expression, we, therefore, set out to investigate whether disruption of the MCT-Basigin interaction may be achieved with a small molecule. Here, we report on the identification of the small molecule Acriflavine as a potent inhibitor of the MCT4 – Basigin interaction, cancer stem-like fraction, and cell growth *in vitro*, and tumor growth *in vivo*. Our work confirms that small molecules can effectively disrupt protein-protein interaction between integral cell-surface proteins *in vitro* and *in vivo* and serve as effective antitumor agents.

## Results

### Screening for inhibitors of MCT – basigin interaction

**Figure 1:**
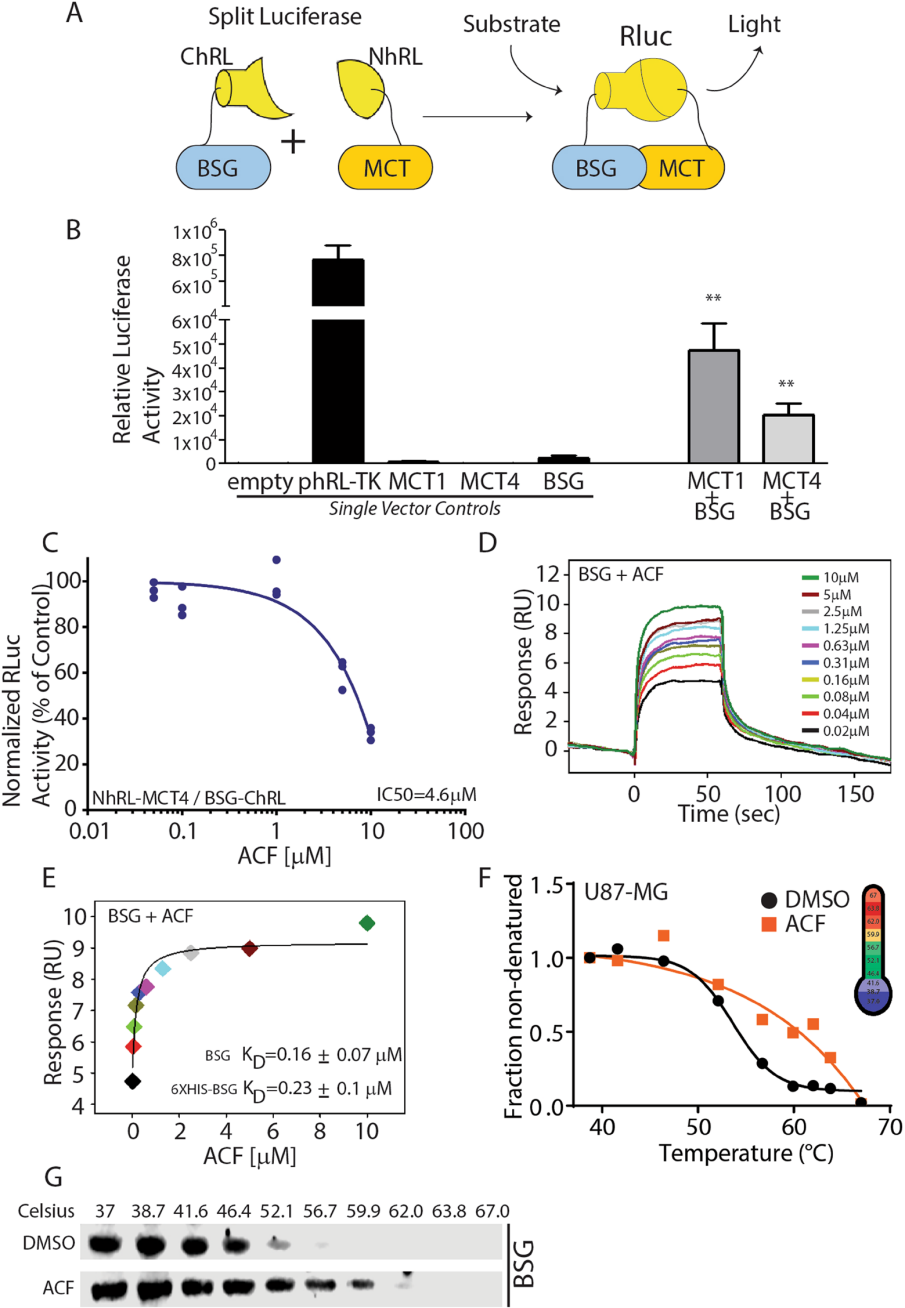
ACF Binds BSG and Inhibits the MCT/BSG Interaction. (**A**) Split Renilla luciferase (Rluc) system for identifying inhibitors of MCT/BSG Interaction. N-terminal and C-terminal portions of Rluc were attached to MCT (MCT1 and MCT4) and BSG, respectively. (**B**) The ratio of Renilla/Firefly luciferase activity (Rluc/Fluc) was determined using HEK293 cells co-transfected with pCDNA3, which encodes Fluc, with the indicated constructs. Each value was then normalized to the results for an empty vector. Data represents mean ± SD (n = 3). **P < 0.01 (Student’s t-test) vs. samples lacking BSG. (**C**) Dose dependency curve for NhRL-MCT4 and BSG-ChRL interaction. IC50 = 4.6 μM. (**D**,**E**) Surface plasmon resonance (SPR) analyses were used to determine the equilibrium dissociation constant (KD) between Basigin and ACF. Sensorgrams (**D**) and a fitting curve (**E**) are shown. Experiments were performed in triplicates and comparison was made between native BSG and 6X-HIS BSG with similar KD values of 0.16+/−0.07 and 0.23 +/− 0.1, respectively. (**F**) A representative cellular thermal shift assay (CETSA) analysis for U87-MG GBM cells treated with ACF or vehicle (DMSO) showing ACF increases BSG’s melting temperature, therefore, providing further evidence for direct drug/target interaction in live cells. (**G**) A representative western blot analysis of CETSA assay.

In this study, we developed a cell-based MCT-Basigin interaction assay utilizing a synthetic Renilla luciferase (Rluc) protein-fragment-assisted complementation-based bioluminescence^24^. Here, the interaction of the full-length MCT1 or MCT4 with full-length Basigin provided the mechanism for complementation (Fig. 1A). Complementation-based restoration of enzyme activity by MCT and Basigin require that the proteins are folded correctly and positioned in close proximity. We transfected vectors encoding NhRL-MCT1, NhRL-MCT4, and Basigin-ChRL fusion proteins into HEK293, and Rluc activity was normalized to Firefly luciferase (FFluc) constitutively expressed from a CMV promoter. Robust Rluc activity was detected in cells expressing NhRL-MCT1 or NhRL-MCT4 and Basigin-ChRL, but not when either NhRL-MCT1, NhRL-MCT4, or Basigin-ChRL were expressed alone (Fig. 1B). Attempts to reconstitute Rluc activity with NhRL fused to the cytoplasmic tails of MCT1 (59aa) or MCT4 (60aa) with the ChRL fused to the cytoplasmic tail (41aa) of Basigin were unsuccessful (data not shown). These results are in agreement with previously published work showing the transmembrane domain of Basigin is required for the interaction with MCT1^23, 25^ and suggest our assay is suitable for identifying inhibitors of MCT – Basigin interaction.

We screened 727 drugs with known biological targets (NIH clinical collections 1 and 2) and identified 8 compounds (~1.1%) that inhibited MCT4-Basigin dependent Rluc activity by ≥40% at a concentration of 10 μM. These hits were subjected to secondary screening using the split Rluc assay. One of the most potent inhibitors of the MCT4 - Basigin interaction was the acridine dye ACF (ACF), which inhibited MCT4-Basigin dependent Rluc activity in a dose-dependent fashion with an IC50 of 4.6 μM (Fig. 1C). The IC50 for ACF inhibiting NhRL-MCT1/ Basigin-ChRL interaction was calculated to be ~19.5 μM, over fourfold higher (Supplemental Figure S1).

### ACF binds directly to the extracellular immunoglobulin domain of basigin

To determine if ACF binds directly to Basigin, we subjected either the native or a 6-His tagged version of Basigin’s extracellular immunoglobulin domain to surface plasmon resonance (SPR) analysis (Fig. 1D,E). The K_D_ values for ACF binding to the native domain or the 6-His tagged domain were calculated to be 0.16 +/− 0.07 μM and 0.23 +/− 0.1 μM, respectively.

### ACF binds to basigin in live glioblastoma cells

Given that ACF binds directly to the purified basigin protein we next sought to determine its ability to bind Basigin in intact live glioblastoma cells. To this end, we performed cellular thermal shift assays (CETSA) in U87-MG glioblastoma cells. The rationale for using this established GBM cell line is that it expresses both Basigin and MCT4 constitutively in normoxia. Basal constitutive expression of the two interacting proteins simplifies the experimental procedure significantly. In this assay, binding of a chemical agent (ACF) to a protein is expected to result in thermal stabilization (or destabilization) of the protein^26^. U87-MG cells with or without treatment with ACF were heated to different temperatures and then extracted in the presence of NP-40 (Fig. 1F). Following data normalization and non-linear curve fitting, we calculated the melting temperature (T_m_) of basigin to be 54.12 °C in DMSO-treated cells. ACF treatment increased Basigin’s T_m_ to 59.50 °C. A representative western blot analysis is shown in Fig. 1G. Taken together, SPR and CETSA assays strongly suggest that ACF binds directly to the Basigin protein in purified form and in live GBM cells. Also, these results provide a potential mechanistic explanation for the results of our split Renilla luciferase assay as they suggest that ACF may inhibit the MCT4-Basigin interaction by directly binding to Basigin’s extracellular immunoglobulin domain and promoting a conformational change leading to the dissociation of the two proteins.

### MCT1 is the primary driver of lactate secretion in GSC

**Figure 2:**
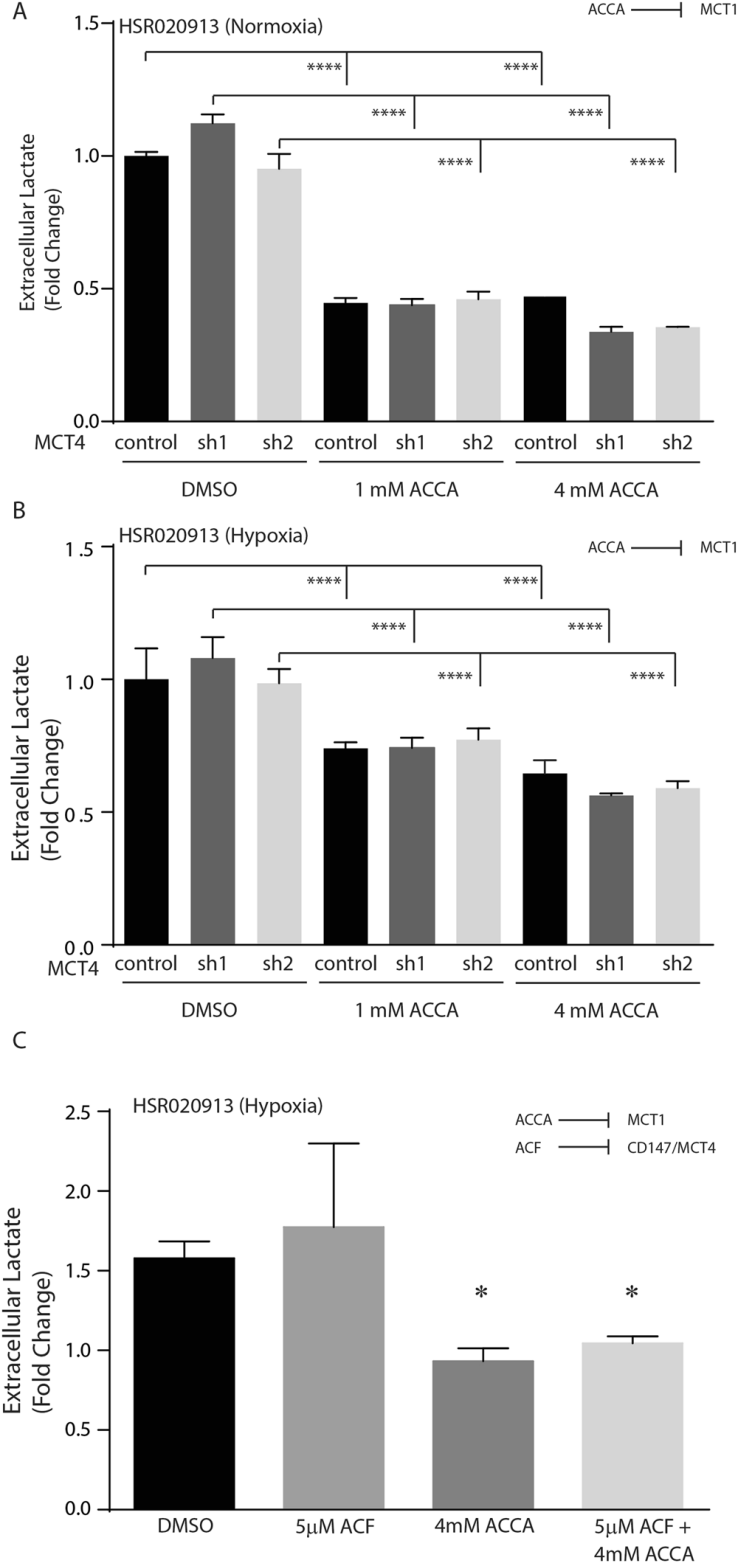
Lactate homeostasis is primarily a function of MCT1 in GSCs. HSR-GBM1, expressing control, shMCT4 sh1 (474) or sh2 (477) were treated with 0 (DMSO), 1 mM or 4 mM Cinnamic acid (ACCA) for 48 hours in normoxia (**A**) or Hypoxia (**B**). (**C**) Examination of extracellular lactate levels in HSR-GBM1 treated in hypoxia for 24 hours with DMSO, 5 μM ACF, 4 mM ACCA, or with the combination of ACF and ACCA. (One-way ANOVA (**A**,**B**) and Student’s t-test (**C**); *p < 0.05, ***P < 0.001, ****P < 0.0001).

We have previously shown that reduced MCT4 expression, by shRNAs, significantly inhibited proliferation and induced apoptosis in GSCs and that these effects were not due to reduced capacity to secrete lactate^9^. Given that, at the concentration we used in our *in vitro* experiments, ACF appears to primarily disrupt the interaction between MCT4 and Basigin but not the interaction between MCT1 and Basigin (Fig. 1C and Supplemental Figure S1), this gave us a unique opportunity to compare the relative roles of the two MCTs in lactate transport in GSCs. To this end, we treated HSR-GBM1 GSC expressing control or shRNAs against MCT4, with 0, 1 or 4 mM Cinnamic acid (ACCA, a specific inhibitor of MCT1) in normoxia and hypoxia and measured extracellular lactate levels as the readout for MCT activity (Fig. 2). Consistent with our previous report^9^, we found that reduction in MCT4 levels did not alter extracellular lactate levels in normoxia (Fig. 2A) or hypoxia (Fig. 2B). Treatment with 1 or 4 mM Cinnamic acid (inhibiting MCT1), significantly reduced extracellular lactate levels from approximately 5 mM to 2.2 mM and 1.9 mM, respectively in normoxia (approximately 65% reduction, Fig. 2A) and to 3.6 mM and 2.84 mM in hypoxia (approximately 42% reduction, Fig. 2B). We next used ACF’s ability to disrupt the MCT4 – Basigin interaction to examine its effects on lactate transport in hypoxia. We quantified extracellular lactate levels in HSR-GBM1 treated with DMSO, 5 μM ACF, 4 mM ACCA, or the combination of ACF and ACCA for 24 hours in normoxia or hypoxia. We found that ACF treatment had no significant effect on the hypoxia-dependent increase in lactate production and secretion as the relative extracellular lactate levels increased by 1.58-fold and 1.77-fold in DMSO and ACF-treated GSCs, respectively. In contrast, inhibition of MCT1 activity, using ACCA, significantly blocked hypoxia-induced lactate secretion as extracellular lactate levels were almost indistinguishable (0.93-fold) in normoxic and hypoxic GSCs, treated with 4 mM ACCA, and reduced by 41% in comparison to DMSO-treated GSCs (p = 0.021, two-sided student’s t-test). Similarly, co-treatment with ACF and ACCA also resulted in 41% reduction in lactate secretion (p = 0.023, two-sided student’s t-test). Taken together, these data confirm that MCT4 inhibition by shRNAs (reduced expression) or by ACF (disruption of the MCT4-Basigin interaction) have no effect on lactate production and secretion and that MCT1 is the primary route by which lactate is transported by GSCs (Fig. 2C).

### ACF inhibits hypoxia-Inducible factor-1 transcriptional activity

Lee and co-workers have previously shown that ACF inhibits HIF-1 DNA-binding and transcriptional activity^27^. We have recently shown that inhibition of MCT4 expression, using short hairpin RNAs, diminishes HIF-1 transcriptional activity in GSCs, suggesting MCT4 plays a role in a feedback mechanism required for maintaining HIF-1 transcriptional activity^9^. To determine to what extent ACF treatment affects HIF-1 transcriptional activity in GSCs, we treated HSR-GBM1 and HSR040622 neurospheres with 0 or 5 μM ACF in normoxia and hypoxia. We then calculated the relative hypoxic induction of three well established HIF-1 target genes: carbonic anhydrase-9 (CAIX), N-Myc Down-Regulated Gene-1 (NDRG1), and Vascular Endothelial Growth Factor (VEGF). We found that treatment with ACF blocked the hypoxic induction of these genes with CAIX reduced from 2564.32-fold and 295-fold to 0.98-fold and 0.96-fold, in ACF-treated HSR-GBM1 and HSR040622, respectively. Similarly, induction of NDRG1 was also blocked from 4261.62-fold, and 8.86-fold to 3.74-fold and 2.73-fold, in ACF-treated HSR-GBM1 and HSR040622, respectively. In addition, VEGF expression was also reduced from 109.05-fold, and 10.14-fold to 1.7-fold, in ACF-treated HSR-GBM1 and HSR040622, respectively. It has been previously shown that MCT4 expression is tightly regulated by HIF-1 activity. Therefore we examined the effects of ACF on MCT4 expression. We found that while hypoxia induced MCT4 expression by over 60-fold, ACF treatment significantly inhibited this induction to 20-fold, an over 60% reduction (Supplemental Figure S2). Together, our results suggest that ACF is an effective inhibitor of HIF-1 transcriptional activity in hypoxic GSC.

### ACF inhibits GSC growth *in vitro*

**Figure 3:**
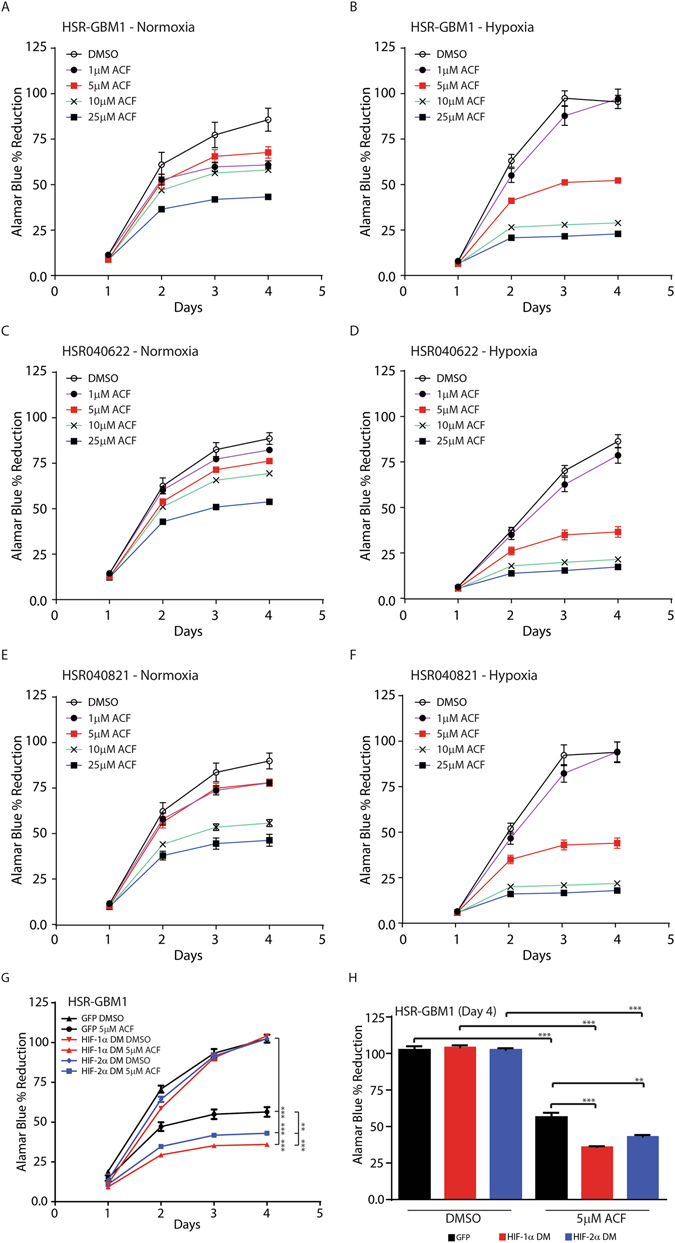
ACF inhibits GSC growth in a dose-dependent fashion, and its anti-growth effect is augmented by hypoxia or by expression of, double-mutant, oxygen stable, forms of HIF-1α or HIF-2α. Growth curves of HSR-GBM1 (**A**,**B**), HSR040622 (**B**,**C**), and HSR040821 (**E**,**F**) treated with 0 (DMSO), 1, 5, 10, and 25 μM ACF in normoxia (21% oxygen; **A**,**C**,**E**) and hypoxia (1% oxygen; **B**,**D**,**F**). (**G**,**H**) Growth curve for HSR-GBM1 GSCs expressing GFP (as control) or the oxygen stable, double mutant (DM), forms of HIF-1α or HIF-2α, and treated with ACF at 0 (DMSO), 1 μM or 5 μM significantly reduced cell growth over a four-day period in normoxia. (H) A bar-graph analysis of day 4 time point is shown in (**G**). (***p < 0.001, **p < 0.01; One way ANOVA with multiple comparisons; Mean +/− SEM of triplicates is shown in all analyses).

We next examined whether ACF may affect the growth of cultured GSC *in vitro*. To this end, three GSC lines, HSR-GBM1, HSR040622, and HSR040821, were cultured in normoxia or hypoxia and treated with 0-25 μM ACF. Total viable cell mass was quantified by an Alamar Blue cell viability assay. We found that treatment with ACF inhibited GSC growth in a dose-dependent fashion. Furthermore, in all three GSC lines, ACF treatment was significantly more effective in slowing cell growth under hypoxia (Fig. 3A–F) highlighting the requirement for the MCT4 – Basigin protein –protein interaction for growth under low-oxygen conditions. To further examine the requirement for the MCT4 – Basigin interaction, we transduced retroviral vectors encoding oxygen-stable^28^ (double mutant – DM) forms of HIF-1α and HIF-2α into HSR-GBM1. We have previously shown that the oxygen-stable form of HIF-1α promotes the transcriptional response to hypoxia under conditions where oxygen is plentiful^3^. We found that ACF-dependent blockade of the MCT4 – Basigin interaction produced significantly larger growth inhibitory effects in GSCs expressing the oxygen-stable forms of HIF1α and HIF2α over a four day period (Fig. 3G). A bar graph analysis of the data for day four is shown in Fig. 3H. Together, our results suggest ACF antitumor activity is augmented by hypoxia and also by synthetic activation of the transcriptional response to hypoxia which is mediated by HIFs.

### ACF inhibits clonogenic capacity *in vitro*

**Figure 4:**
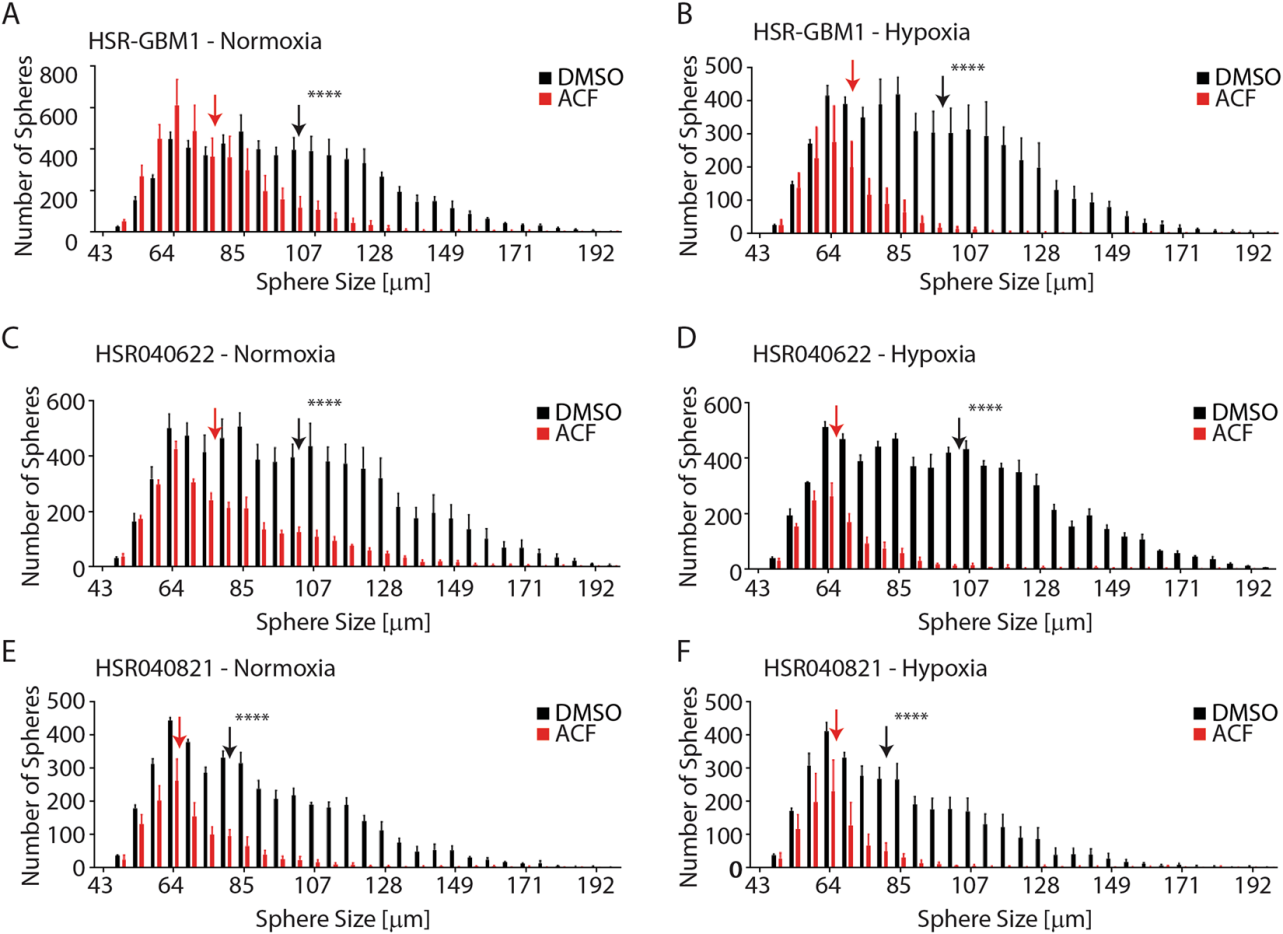
Clonogenic analysis in NSMC medium. HSR-GBM1 (**A**,**B**), HSR040622 (**C**,**D**), and HSR040821 (EF) were treated for 72 hours with ACF (5 μM) or vehicle (DMSO) in normoxia (**A**,**C**,**E**) or hypoxia (**B**,**D**,**F**). Following 24 hours of recovery (in normoxia and without drug), cells were plated in NSMC medium. The clonogenic potential of ACF-treated GSCs was significantly lower as compared with vehicle in each of three GSC lines. (****p < 0.0001, One way ANOVA with multiple comparisons).

We next sought to determine if ACF may reduce GBM clonogenic potential. We have previously demonstrated that MCT4 promotes proliferation, survival and xenograft implantation/growth of several GSC neurosphere lines^9^. Also, we showed that cultured GBM cells form large neurospheres in methylcellulose substrate (NSMC) only when initiated by GSC but not by more differentiated progenitors^3^. Therefore, we treated HSR-GBM1, HSR040622, and HSR040821 neurosphere-derived cells with 5 μM ACF or vehicle (DMSO) and incubated in normoxic or hypoxic conditions for 72 hours. Next, the drug was removed, and the cells were allowed to recover, in normoxia, for 24 hours. An equal number of viable cells were then seeded in methylcellulose substrate (NSMC) and grown for nine days under normal oxygen tension. Triplicate wells for each growth condition were photographed, and the overall number and the diameter of the neurospheres were measured. We found that treatment with ACF reduced the clonogenic potential of all GSC neurosphere lines we tested and these anti-GSC effects were augmented by hypoxia (Fig. 4). ACF treatment decreased the average sphere diameter from 96.54 μm to 74.37 μm (P < 0.0001) for normoxic HSR-GBM1 (Fig. 4A) and from 96.54 μm to 67.76 μm for hypoxic HSR-GBM1 (P < 0.0001) when compared to vehicle-treated cells (Fig. 4B). Similarly, in HSR040622, average sphere diameter was decreased by ACF treatment from 97.44 μm to 80.77 μm (P < 0.0001) for normoxic HSR040622 (Fig. 4C) and from 98.77 μm to 68.10 μm for hypoxic HSR040622 (P < 0.0001) (Fig. 4D). Finally, treatment with ACF decreased the average sphere diameter from 88.75 μm to 69.92 μm (P < 0.0001) for normoxic HSR040821 (Fig. 4E) and from 83.35 μm to 66.49 μm for hypoxic HSR040821 (P < 0.0001) when compared to vehicle-treated cells (Fig. 4F).

### ACF inhibits GSC-derived xenograft growth and tumor vascularization

**Figure 5:**
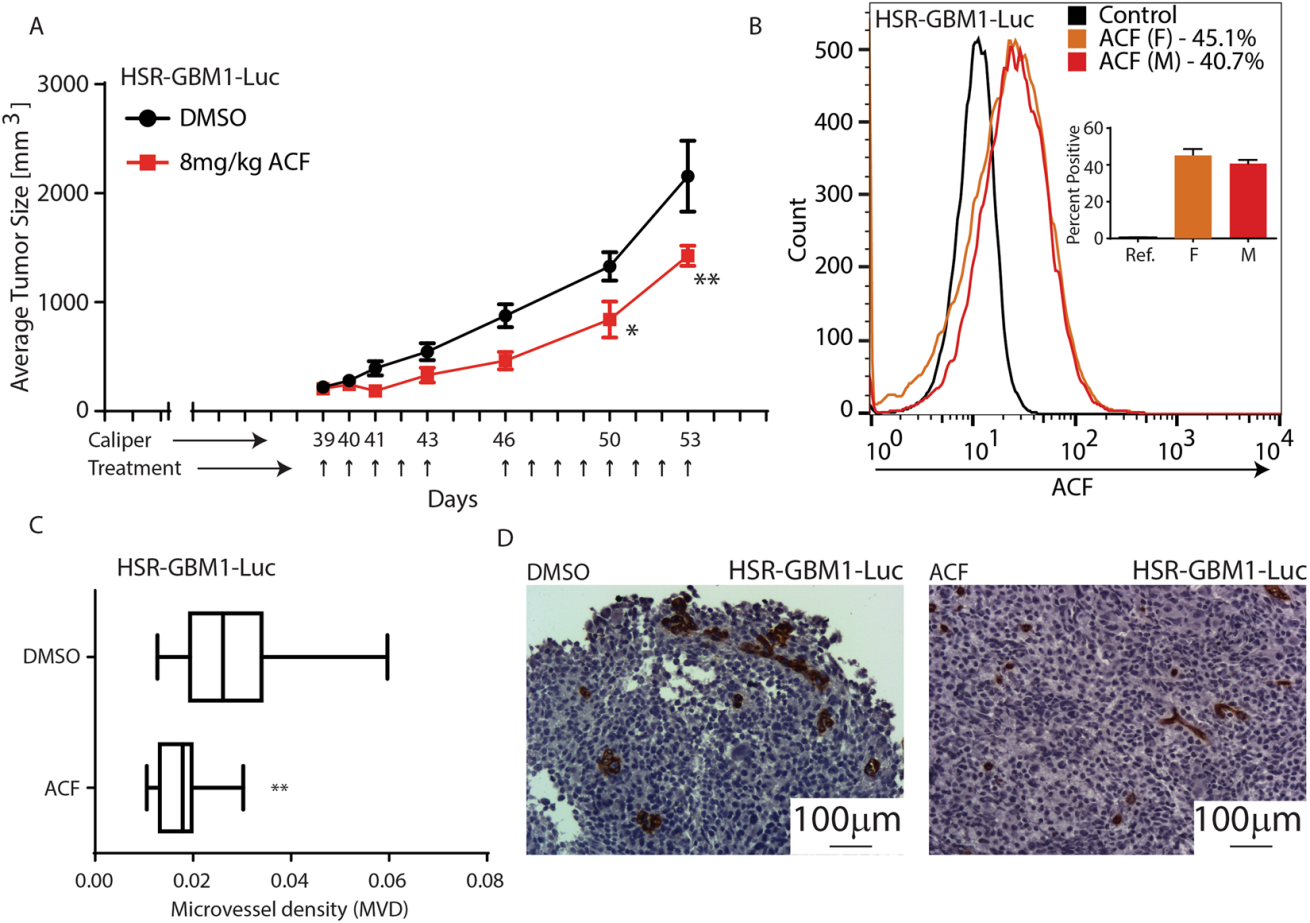
ACF inhibits HSR-GBM1-Luc xenograft growth *in vivo*. (**A**) HSR-GBM1 xenografts were grown to approximately 220 mm^3^. Treatment was administered by intraperitoneal injections of vehicle (DMSO in saline) or ACF (8 mg/kg) for 15 injections (indicated by arrows in (**A**)). Tumor volume (mean +/− SEM; n = 5) is shown. (**B**) To determine if ACF is reaching its target *in vivo*, two mice (one female and one male) were euthanized on day 41 (Third daily injection and 2 hours after the last dose) and their tumors were harvested and analyzed by flow cytometry, with HSR-GBM1-Luc serving as a reference. (**C**,**D**) Microvessel density analysis for xenografts shown in (**A**) using IHC staining for CD31; **P < 0.01 ACF vs. vehicle (two-way ANOVA with Sidak’s multiple comparisons test).

We next examined if ACF may inhibit the growth of (1) large xenografts (average size approximately 220 mm^3^; 39 days post tumor cell injection), and (2) small xenografts when tumors were detectable by bioluminescence imaging but not yet palpable (16 days post tumor cell injection). In this study, we employed heterotopic (flank) and not orthotopic (brain) as it has been previously shown that in otherwise identical GSC models, flank xenografts exhibit significantly higher levels of hypoxia and necrosis. In the first experiment, large xenografts, vehicle (n = 9 tumors) or ACF (n = 7 tumors) was administered by intraperitoneal injection (Fig. 5A). The duration of the experiment had to be limited primarily due to the size of the tumors in the control group which grew to approximately 2150 mm^3^ by day 53. Tumors in mice treated with ACF grew to approximately 1420 mm^3^. Importantly, administration of ACF did not cause significant weight loss, and most animals continued to gain weight over the duration of the experiment (Supplemental Figure S3A), suggesting ACF has no major systemic toxicity at the dose we used. We next took advantage of the fluorescent properties of ACF to determine if, and to what extent, ACF was taken up by tumor cells. To this end, we sacrificed two animals (one male and one female), each carrying a single xenograft, on the third day and two hours after the last ACF injection. Xenografts were immediately processed to obtain single-cell suspensions that were analyzed by flow cytometry. We found that 40–45% of the cells extracted from ACF-treated xenografts were brightly fluorescent in comparison to HSR-GBM1-Luc cells set as a reference (Ref.; Fig. 5B). To determine if ACF affects tumor vascularization, we calculated microvessel density (MVD) in the vehicle and ACF-treated tumors by immunohistochemistry. We found that ACF reduced tumor microvessel density from an average density of 0.028 ± 0.0026 (n = 18) to 0.0173 ± 0.0011 (n = 20) (Fig. 5C, t-test with Welch correction, **p = 0.0011). A representative image from each group is shown in Fig. 5D. Collectively, these results suggest that systemically administered ACF is taken up by tumor cells, inhibits tumor vascularization, and significantly slows the growth of large GSC-derived tumors.

**Figure 6:**
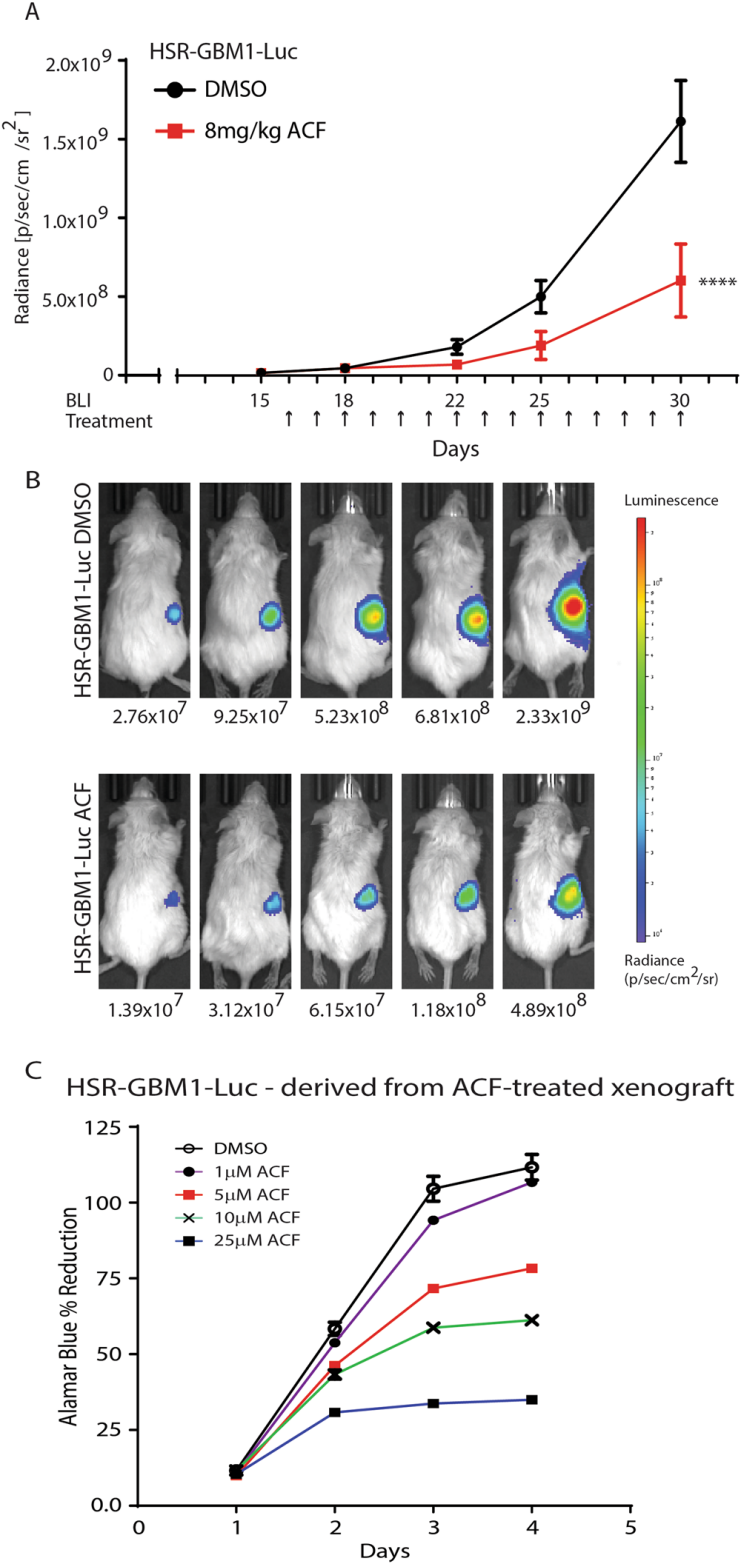
ACF inhibits the growth of small, visually undetectable, HSR-GBM1-Luc xenograft growth *in vivo*. (**A**) HSR-GBM1-Luc xenografts were grown for 15 days. Treatment was administered by intraperitoneal injections of vehicle (DMSO in saline) or ACF (8 mg/kg) for 15 injections (indicated by arrows in (**A**)). Averaged luciferase activity (radiance) is shown. ****p < 0.0001 ACF vs. vehicle (two-way ANOVA with Sidak’s multiple comparisons test). (**B**) BLI of representative (of the mean) animals are shown for the vehicle (top) and ACF-treated animals (bottom). (**C**) Alamar Blue *in vitro* growth assay was performed on HSR-GBM1-Luc cells freshly isolated from ACF-treated animals. Cells were treated in hypoxia (1% oxygen) with 0 (DMSO), 1, 5, 10, and 25 μM ACF. A representative analysis is shown.

In small xenografts, the integrated luciferase gene allowed us to generate equivalent treatment groups and facilitate tracking of xenografts. We followed a similar treatment schedule as described above (Fig. 6A) with the exception that tumor monitoring was performed by bioluminescence imaging. ACF antitumor activity was noticeable as early as day 22 (seventh injection). This difference became strongly significant on day 30 with tumors showing an average radiance (p/sec/cm^2^/sr) of 1.61 × 10^9^ and 6.05 × 10^8^ in tumor-bearing animals treated with vehicle and ACF, respectively (two-way ANOVA, ****p < 0.0001 with Sidak’s multiple comparisons test) (Fig. 6A,B). Following the last bioluminescence imaging, (day 30) we attempted to excise all tumors. Strikingly, while all vehicle-treated animals had large xenografts that were easily dissected, in 3 of 10 ACF-treated animals, tumors regressed to the point that no dissectible mass could be found. Finally, ACF administration did not cause any significant loss of body weight (Supplemental Figure S3B).

### GSC-derived from ACF-treated tumors remains sensitive to the drug when challenged *in vitro*

Because drug resistance may develop while tumors are under pressure of treatment, we next determined if inhibition of the MCT4-Basigin interaction *in vivo* may promote resistance. To this end, we derived GSCs from DMSO and ACF-treated animals. We then challenged these cells with different concentrations of ACF for 96 hours *in vitro* and measured viable cell mass by an Alamar Blue assay. GSCs from ACF-treated HSR-GBM1-Luc xenografts remained sensitive to ACF *in vitro* in hypoxia, similar to the parental GSC line shown in Fig. 3B, suggesting that the period of *in vivo* therapy had not resulted in broad resistance to MCT4-Basigin inhibition (Fig. 6C). Taken together, our *in vivo* studies show that ACF is an effective agent inhibiting GSC-derived tumors and that the mechanism against GSCs is likely to involve inhibition of the MCT4-Basigin interaction as well as inhibition of the hypoxic response.

## Discussion

Altered metabolism with high rates of glucose consumption and, as a consequence, high levels of lactate is one of the recently recognized hallmarks of cancer. To facilitate this phenotype, many cancers efficiently upregulate the expression of a large number of cell surface transporters, including MCTs. As lactate/proton symporters, MCT1 and MCT4, achieve two important goals at once. They transport the lactate produced following the reduction of pyruvate, and for every molecule of lactate, they also eliminate a proton, therefore assist in maintaining a basic intracellular environment which is required for many cellular functions to proceed. While the historical view of lactate has been, for the most part, as a by-product of glycolysis, more recent studies^29, 30^ have clearly demonstrated that lactate is a key player in cancer, regulating processes such as tumor cell proliferation, angiogenesis, invasion, and reduced cytotoxic T-Cell activity. Furthermore, high extracellular lactate levels have been linked to tumor resistance to radiation therapy and to poor patient survival. (recently reviewed by^30^). These functions, among others, have made MCTs attractive targets in cancer therapy, especially in cancers with a hyper-glycolytic and acid-resistant phenotypes, such as GBM.

In previous reports, we showed that the proliferation and survival of GSC are dependent on hypoxia inducible factor-1α^3, 8^, the master regulator of the transcriptional as well as metabolic reprogramming of hypoxic cells. In a more recent report we identified hypoxia-induced MCT4 as a key regulator of HIF-1α in multiple GSC neurosphere models^9^. Recent reports established that the cell surface protein Basigin is a chaperone for both MCT1 and MCT4. Because this protein-protein interaction between Basigin and MCT1 or MCT4 appears to be an absolute requirement for their proper plasma membrane localization, this prompted us to hypothesize that a small molecule, capable of disrupting this interaction, may possess strong antitumor effects by targeting, among others, GSCs.

We used a cell-based split-Rluc assay drug screen and identified ACF as a drug that inhibits the MCT4 – Basigin interaction by binding to Basigin’s immunoglobulin-like extracellular domain (Fig. 1). Because ACF interacts directly with Basigin, but significant inhibition of the split luciferase reporter occurs only when MCT4 is used as bait, we propose, that MCT1 – Basigin interaction may require different and additional residues. Therefore, the conformational change in Basigin, imposed by ACF, may disrupt MCT4’s but to a lesser extent MCT1’s ability to interact with Basigin. In-depth structure-function studies will be required to define critical residues involved in ACF binding and its effects on MCT – Basigin interactions.

ACF’s anti-cancer activity has been previously reported in several studies^27, 31–34^. Potentially most relevant to our current study is the report by Lee and coworkers showing that ACF inhibits HIF dimerization by binding to the PAS domain of the proteins^27^. While we do not exclude the possibility that some of the effects we report here are mediated by disruption of HIF dimerization, we believe ACF activity in disrupting MCT4-Basigin interaction plays an important role. We base this on our previously published work and the results we obtained in the current study where we showed that inhibition of MCT4, using shRNAs^9^ or ACF (Fig. 3A–F), resulted in decreased growth and clonogenic potential of several GSC neurosphere lines *in vitro*. Most importantly, and consistent with the *in vitro* growth assays, ACF anti-GSC activity was augmented by hypoxia or synthetic activation of the transcriptional response to hypoxia, orchestrated by HIFs (Figs 3 and 4). We suggest that ACF activity is mediated, at least in part, by inhibition of the MCT4 – Basigin interaction and that the reduction in HIF-1 transcriptional activity is primarily the result of reduced MCT4 activity. This is supported by the observation that ACF treatment of GBM neurospheres is augmented when oxygen-stable and non-degradable forms of HIF-1α or HIF-2α are expressed (Fig. 3G,H). We have previously shown that expression of an oxygen-stable form of HIF-1α significantly negated the effects of the HIF1-α translation-inhibitor Digoxin^3^. Therefore, we reason that if the ACF effects were primarily due to disruption of HIF dimerization, overexpression of HIF-1α or HIF-2α should have “rescued” ACF activity, potentially by increasing the absolute amount of “active” HIF heterodimers as HIF1β (ARNT) is never limiting^35–38^. It is also important to emphasize that while it is clear that ACF inhibits MCT and Basigin interaction, ACF inhibition of HIF-1 represents a second point of engagement for ACF in inhibiting the hypoxic response. Therefore it is not surprising that we found the tumors treated with ACF *in vivo* did develop resistance to ACF as the ability of cells to develop resistance to two points on engagement would be considered to be very unlikely.

We chose to engraft our GSCs into flank rather than brain primarily due to the fact that GSCs implanted in the flank consistently show higher levels of hypoxia/necrosis as compared to xenografts grown in the brain (ref. 39, and data not shown). Also, the main focus of the current study is an exploration of the effects of disruption of the MCT-Basigin protein-protein interaction. We suggest that further studies will be required to investigate the ability of ACF to cross the blood-brain-barrier.

In our xenograft studies, we aimed to determine if ACF could effectively inhibit tumor growth of established large as well as small tumors. Daily intraperitoneal treatment with ACF was effective in inhibiting the growth of xenografts generated from the temozolomide resistant^40^ neurosphere line HSR-GBM1 as measured by bioluminescence in serial imaging. Using flow cytometry, we could readily detect ACF in tumor cells as early as two hours following drug administration. Further supporting the notion that ACF reaches tumor cells are the quantitative real-time PCR analyses of tumor tissues that showed decreased expression of some HIF-1 targets indicating that ACF may effectively inhibit MCT4-Basigin interaction *in vivo*. As a side note, fluorescence detection (using BD IVES Spectrum) in live animals, treated with 8 mg/kg ACF, showed that six hours from drug administration most of the fluorescent signal was concentrated in the bladder confirming renal excretion as the major path for ACF elimination (Data not shown).

We are encouraged by the observation that prolonged ACF treatment *in vivo* did not promote a broad resistance to ACF, as GSCs isolated from drug-treated xenografts remained sensitive to ACF with similar IC50 (~5 μM). Our co-treatment studies strongly suggest that ACF therapy will greatly benefit from a combinatorial approach when combined with radiation in a future clinical setting if long-term survival of GBM patients is to be achieved.

In summary, the need to target multiple pathways for effective GBM therapy is becoming accepted. High-throughput profiling of GBM by The Cancer Genome Atlas (TCGA) made clear that each GBM has lesions in multiple cancer-related pathways^41^, and other reports have also underscored the need to inhibit multiple pathways for GBM therapy. One potential approach lies in the targeting of signaling nodes necessary for numerous critical cancer pathways using single agents, such as ACF and combining them with ‘standard of care’. In this report, we focused on hypoxia-induced MCT4 and its chaperone Basigin (CD147) as a signaling node in the response of GBM to hypoxic stress. We identified the small molecule, ACF, as an effective agent that significantly disrupts the MCT4-Basigin interaction and the transcriptional activity of HIF-1α. As a consequence of these activities, ACF inhibits clonogenic potential and the proliferation of temozolomide resistant and sensitive GBM stem-like cells *in vitro* and *in vivo*. We believe that therapies that are directed at specific mechanisms of brain tumor progression, such as the cellular response to hypoxia, offer the best hope for improved patient survival.

## Materials and Methods

### GBM neurosphere lines and hypoxic conditions

HSR-GBM1 (020913), HSR040622 (040622), and HSR040821 (040821) were a kind gift from Dr. Angelo Vescovi, Milan, Italy. Each line was authenticated by STR analysis once each year in the originator laboratory. A large batch of each line was generated and frozen down in liquid nitrogen for future studies. Once thawed, the lines were maintained in culture as originally described^42^. Subclass determination confirmed these lines primarily express a proneural and classical signatures. A hypoxic chamber maintained at 37 °C, 1% O_2_, 5% CO_2_ and 94% N_2_ (COY laboratory equipment, Grass Lake, MI, USA) were used to conduct *in vitro* hypoxic experiments. All hypoxic experiments were conducted on cells that were plated and allowed to recover overnight before hypoxic induction. We use the term glioblastoma stem cells (GSC) interchangeably with glioblastoma/GBM neurospheres. These cultures are enriched for stem-like glioblastoma cells shown to differentiate into the three neural lineages *in vitro* given the appropriate stimulus (see ref. 42 for details).

### Expression and purification of the Basigin/CD147 immunoglobulin extracellular domain

A derivative of plasmid pET15b encoding the extracellular domain of Basigin/CD147 (residues 22–205) with an N-terminal hexahistidine tag followed by a tobacco etch virus (TEV) protease cleavage site was a gift from Matthew Higgins (Oxford University, UK). In the protein expressed from this plasmid, an extra glycine remains at the N-terminus after cleavage by TEV protease. The protein was expressed in *E. coli* strain Origami B (DE3) (Novagen) as described in^43^. The extracellular domain was purified by nickel-nitrilotriacetic acid (Ni2+−NTA; Qiagen) affinity chromatography. For removal of the 6-His tag, the buffer was exchanged with PBS using dialysis, followed by overnight cleavage at 4 °C. The cleaved affinity tag was removed by a second step of affinity chromatography.

### RNA, DNA, and protein analyses

RNA analyses were performed by reverse transcription into complementary DNA and then quantitative real-time PCR analysis in triplicates using SYBR Green reagents (Bio-Rad, Hercules, CA). To prevent amplification of genomic DNA, on-column DNase digest was performed (ZYMO Research, Irvine, CA) during RNA extraction prior to reverse transcription. Standard curve analyses were used to determine RNA levels, and RPLP0 was used as a housekeeping gene for normalization. Primer sequences were as follows: MCT1 (SLC16A1) forward—5′- CCATTGTGGAATGCTGTCCT-3′ and reverse—5′- CCACATGCCCAGTATGTGTA-3′; MCT4 (SLC16A3) forward—5′-GAGTTTGGGATCGGCTACAG-3′ and reverse—5′-CGGTTCACGCACACACTG-3′; CAIX forward—5′-CTTGGAAGAAATCGCTGAGG-3′; and CAIX reverse—5′-TGGAAGTAGCGGCTGAAGTC-3′. Additional primer sequences are available upon request.

HA-HIF1alpha P402A/P564A-pBabe-puro and HA-HIF2alpha-P405A/P531A-pBabe-puro were a gift from William Kaelin (Addgene plasmids # 19005 and #19006, respectively).

Protein analyses were performed via immunoblot assays as previously described^44^. Antibodies used were: MCT4 (sc-50329, 1:250, Santa Cruz Biotechnology, Santa Cruz, CA), HIF1α (Catalog number 610959, 1:250, BD Biosciences, Franklin Lakes, NJ, USA) and β-Tubulin (Catalog number mAB5564, 1:10,000, Millipore, Billerica, MA).

### Determination of the ACF dissociation equilibrium constant (KD) by surface plasmon resonance (SPR)

Surface plasmon resonance (SPR) analyses were performed using a BIAcore T200 instrument (GE Healthcare, Marlboro, MA). A blank immobilization was done with ethanolamine as controls on flow channels 1 and 3. The purified Basigin/CD147 immunoglobulin extracellular domain was diluted to 50 µg/mL with 10 mM sodium acetate (pH 4.0) and injected for 20 min at a 7 µL/min flow rate on a CM5 sensor chip (Series S Sensor Chip CM5; GE Healthcare) for immobilization using standard amine coupling, at 25 °C, with running buffer PBS-P (10 mM phosphate pH 7.4, 2.7 mM KCl, 137 mM NaCl, and 0.05% surfactant P-20). Basigin/CD147 was immobilized in flow channels 2 and 4 after sensor surface activation by a 1-ethyl-3-(3-dimethylaminopropyl) carbodiimide hydrochloride (EDC)/N-hydroxysuccinimide (NHS) mixture. Ethanolamine blocking of the unoccupied surface area was performed at 25 °C. Basigin/CD147 immobilization levels in flow channels 2 and 4 were ~3,500 RU and ~5,200 RU, respectively. ACF solutions with a series of increasing concentrations (0.02–10 µM) were applied to all four channels at a 30 µL/min flow rate with assay buffer (10 mM phosphate, pH 7.4, 2.7 mM KCl, 137 mM NaCl, 0.05% surfactant P-20, 0.5 mM TCEP, and 2% DMSO), and real-time response units (RU) were monitored. Sensorgrams were analyzed using the Biacore T200 evaluation software v3.0. Data were referenced with blank channel RU values, and the KD values were determined by fitting the reference subtracted data to steady-state affinity equation embedded in Biacore T200 evaluation software.

### Cellular Thermal Shift Assays using intact glioblastoma cells (CETSA)

U87-MG cells were cultured in T75 cm^2^ tissue culture flasks until ~80% confluency was reached (density of ~1.5 × 10^5^ cells/cm^2^). Then, growth medium was replaced with 8 ml of complete medium containing either 20  μM ACF dissolved in DMSO or an equivalent amount of DMSO (vehicle). Cells were incubated with ACF and vehicle in parallel for one hour at 37 °C and 5% CO_2_. The cells were pelleted at 360x g at room temperature for 5 min, resuspended in PBS, and centrifuged again as indicated above. This washing step was repeated once, and the cells were carefully resuspended in 200 μl PBS containing protease inhibitor cocktail without EDTA (Roche). 20 μl aliquots of this resulting cell suspension were transferred into ten wells of 96-well PCR plates. The cells were then heated in a preset temperature gradient (37 °C – 67 °C) in a PCR machine (Bio-Rad T-100). Heat was applied for 3 min followed by a 3-min incubation time at room temperature. 30 μl of ice-cold PBS containing protease inhibitor cocktail without EDTA and 0.4% NP-40 detergent (Sigma, to a final detergent concentration of 0.24%). We included detergent during extraction after cell heating as it has been previously shown not to affect protein T_m_ values^26^. Thereafter, the cells were snap-frozen in liquid nitrogen for 1 min, then thawed briefly at 25 °C. This freeze-thaw cycle was repeated once. The entire content was centrifuged at 3160x g for 30 min at 4 °C. After lysate clarification, 20  μl of each supernatant was transferred into new tubes. Proteins in the supernatant were denatured by using SDS sample buffer (Invitrogen, LDS buffer with 50 mM DTT as reducing agent) followed by western blotting and immuno-detection with antibodies against Basigin (1:250, Basigin antibody (8D6): sc-21746, Santa Cruz Biotech, CA). For all CETSA experiments, the intensity of each band (Fig. 1G) was determined by ImageJ. Each band’s intensity was normalized to the lowest temperature which was set as baseline to establish fold-change. Then, using GraphPad version 6 software, we applied the software integrated ‘non-linear regression algorithm’ to calculate Basigin’s melting point (Tm) in DMSO and ACF-treated cells.

### Lactate assays

7 × 10^4^ HSR-GBM1 cells were seeded in phenol-red-free medium and allowed to recover overnight. Cultures were then treated with either 0.1% DMSO, 5 μM ACF, 4 mM ACCA, or 5 μM ACF + 4 mM ACCA combination and placed in normoxia or hypoxia (1% oxygen) for an additional 24 hours. To remove extracellular lactate, cells were rinsed once with fresh treatment-containing phenol-red-free medium and then incubated for 24 hours in normoxia to measure the effect of the treatment on the ability of cells to secrete excess lactate into the culture medium. Extracellular lactate measurement was performed according to the manufacturer’s instructions (L-Lactate Assay Kit, Eton Biosciences, San Diego, CA).

### Treatment of large tumors with ACF

In all experiments involving animals, the experiments were executed in compliance with institutional guidelines and regulations (IACUC #2012–0132 followed by #2015–0100). Male and female NOG mice (NOD.Cg-Prkdcscid Il2rgtm1Wjl/SzJ, The Jackson Laboratory, Bar Harbor, ME) were used in all experiments in approximately equal numbers (+/−1) of each sex in each experimental group. 3.5 × 10^6^ HSR-GBM1-Luc cells were diluted in 200 μL of Modified Hanks’ Balanced Salt Solution (Sigma, #H6648) with sodium bicarbonate, without phenol red, calcium chloride and magnesium sulfate, then mixed lightly with 200 μL of growth factor reduced basement membrane matrix (Corning, #356231) and injected subcutaneously using a 26 G × 5/8 needle (BD, #305115) into the left and right flanks of NSG mice. Xenografts were allowed to develop for five weeks post-implantation at which point all were palpable. Animals were distributed into treatment groups based on tumor size. Animals either received an intraperitoneal injection of a mixture of 0.1% DMSO in saline as a vehicle or 8 mg/kg ACF in saline (Sigma #01673) for a total of 13 injections. Tumor size was measured using a digital caliper (Marathon #CO030300) and tumor volume was calculated as previously described^27^. After sacrifice, flank xenografts were removed and sectioned into three parts. One part was formalin fixed and paraffin embedded for histopathology assays. Another part was snap frozen on dry ice for molecular analyses. The third part was cultured and analyzed by flow cytometry for the presence of ACF using a Guava EasyCyte 5HT with InCyte software version 2.6 (Millipore, Billerica, MA).

### Immunohistochemistry (IHC) and Micro-Vessel Density Quantification

Formalin-fixed and paraffin-embedded (FFPE) tissue specimens were cut into 5-μm sections and placed on StarFrost slides (Thermo Fisher Scientific). The samples were deparaffinized in xylene and rehydrated using a series of graded alcohols. Antigen retrieval was performed by heat in citrate buffer (pH 6.0), followed by 30 min incubation in 3% hydrogen peroxide solution to quench endogenous peroxidases. Samples were blocked with 1% goat serum for 15 min before incubation with primary rabbit monoclonal anti-CD31 antibody (PECAM-1, (D8V9E) XP, Cell Signaling Technology, Danvers, MA) for 45 min. Immunohistochemical staining was performed with a VECTASTAIN Elite ABC HRP Kit (Vector Laboratories, Burlingame, CA), according to the manufacturer’s instructions. Microvessel density was calculated using ImageJ. Ten high-power images were captured using a Zeiss AxioScope A1 equipped with a Zeiss AxioCam105 camera. Each image was processed in the following sequence of steps: (1) the image was converted from Carl Zeiss (*.czi) to RGB format; (2) the total tissue area was calculated using the threshold tool; (3) the total area positive for CD31 was also calculated using the threshold tool; (4) the ratio of CD31-positive to total tissue area was calculated.

### Treatment of small tumors with ACF

Xenografts were established as described above. Two weeks after the injection of cells, animals were distributed into treatment groups based on tumor size which was determined by bioluminescence imaging (IVIS Spectrum system equipped with Living Image software package, Perkin Elmer). Animals either received intraperitoneal injections of a mixture of 0.1% DMSO in saline as vehicle or 8 mg/kg ACF in saline (Sigma #01673) for a total of 15 injections. Tumor size was measured using bioluminescence imaging. After sacrifice, xenografts were removed and sectioned into two parts. One part was snap frozen on dry ice for molecular analyses. The second part was dissociated into single cell suspensions. Roughly one-half was immediately analyzed by flow cytometry to detect the presence of ACF in cells (as described above). The remaining cells were cultured for three days and then challenged with vehicle or ACF *in vitro*. Total viable cell mass was determined by an Alamar Blue growth assay (see below).

### Alamar Blue Growth Assays

GBM stem-like cells were plated at 1500 cells per well of a 96-multiwell plate in 80 μl of neural stem cell growth medium and allowed to recover for 24 hours. Then, 10 μl of either 1% DMSO (as vehicle) or ACF and 10 μl of Alamar Blue reagent (ThermoFisher Scientific catalog #DAL1100) were added to a final volume of 100 μl. Cells were incubated for 4 hours and fluorescence was read using a BioTek Synergy HT equipped with the Gene5 software package (BioTek, Winooski, VT). Daily readings were performed using Excitation 530/25 and Emission at 590/30 filter sets, and analysis of the data was performed according to the manufacturer instructions.

### Clonogenic Assays in NSMC

Clonogenic assays were performed essentially as previously described^3^. Neurosphere number and size were determined using an automatic neurosphere counter GelCount^TM^ (Oxford OPTRONIX, United Kingdom). Data was exported into Microsoft Excel using the propriety software and analysis was performed in GraphPad Prizm Version 6. NSMC = neural stem cell medium containing methylcellulose.

### Statistics

Two-sided t-tests and frequency distributions were performed using GraphPad Prizm software Version 6. Unless otherwise noted, error bars represent SEM for (at a minimum) duplicate experiments.

### Study Approval

Animal studies have been approved by the Case Western Reserve University IACUC committee. Approval #: 2015–0100; Study title: Targeting Brain Tumors in the Hypoxic Microenvironment.

## Electronic supplementary material

supplementary figures
