# Supplementary material for: Disruption of the monocarboxylate transporter-4-basigin interaction inhibits the hypoxic response, proliferation, and tumor progression
Source: Sci Rep. 2017 Jun 27;7:4292. doi: 10.1038/s41598-017-04612-w (PMC5487345; doi:10.1038/s41598-017-04612-w)
Supplement: Supplementary file 1 — supplementary figures [file 41598_2017_4612_MOESM1_ESM.pdf]

Disruption of the monocarboxylate transporter-4-basigin interaction inhibits the hypoxic response, proliferation, and tumor progression – *Supplemental Figures*

Dillon M. Voss[1], Raffaella Spina[1], David L. Carter[1], Kah Suan Lim[2], Constance J. Jeffery[3], and Eli E. Bar[1,*]

[1]Department of Neurological Surgery, Case Western Reserve University School of Medicine and The Case Comprehensive Cancer Center, Cleveland, OH, USA
[2]Department of Radiation Oncology, Dana-Farber Cancer Institute, Boston, MA, USA
[3]Department of Biological Sciences, The University of Illinois at Chicago, Chicago, IL, USA

Conflict of interest statement: The authors have declared that no conflict of interest exists

*** Lead Contact:**
Eli E. Bar, PhD
Assistant Professor, Department of Neurological Surgery, School of Medicine, Case Western Reserve University and The Case Comprehensive Cancer Center, OH
Adjunct Assistant Professor, Johns Hopkins University School of Medicine, MD
Adjunct Staff, Department Stem Cell Biology and Regenerative Medicine, Lerner Research Institute, Cleveland Clinic, OH

Mailing Address:
Case Western Reserve University
Department of Neurological Surgery
Robbins Building, Room E750A
10900 Euclid Avenue
Cleveland OH, 44106-4938
Office (216) 368-0933
Fax (216) 368-1144
E-mail: eli.bar@case.edu

Supplemental Figure S1. VOSS, D.M.

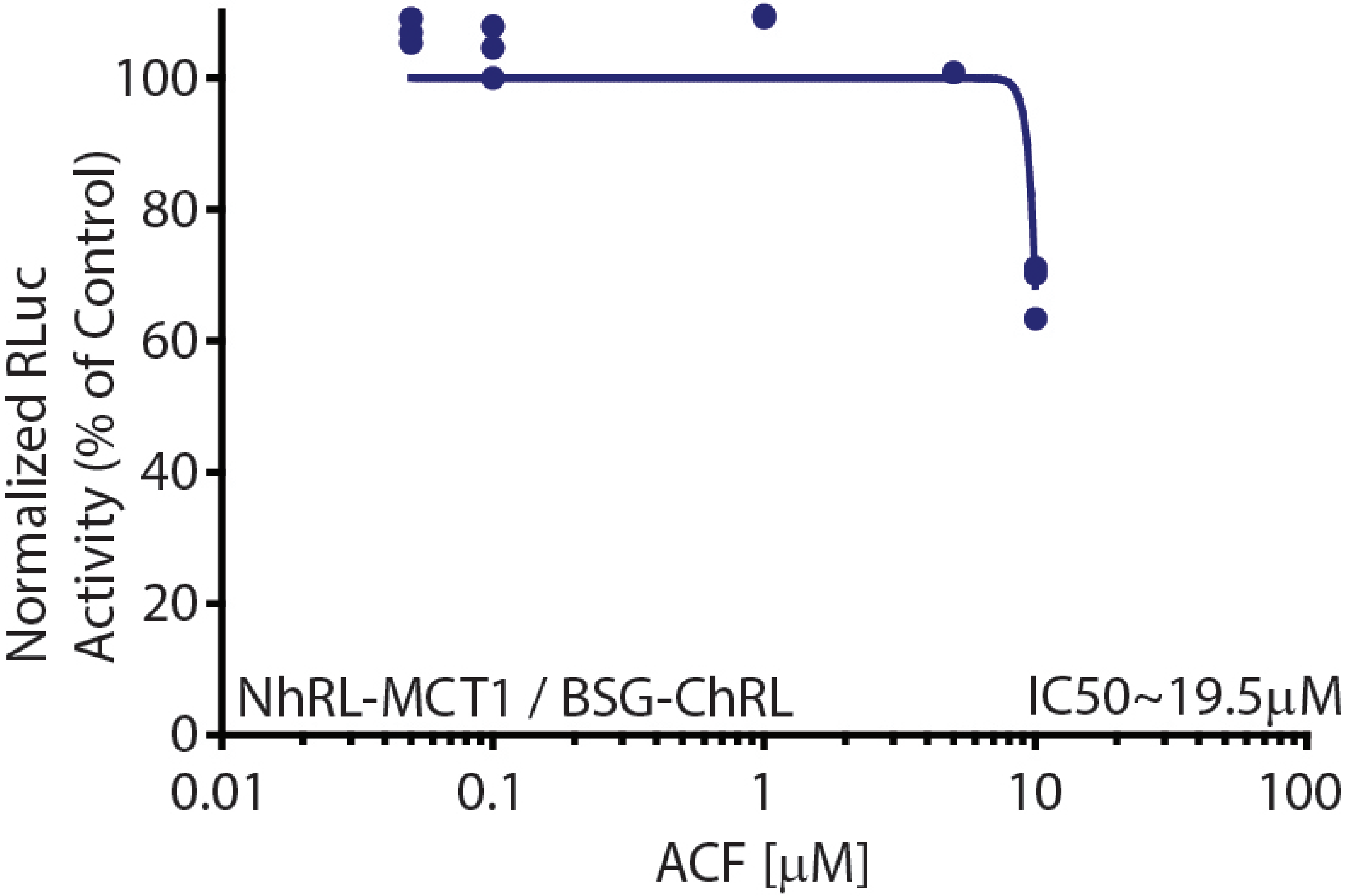

Supplemental Figure S1. Dose dependency curve for NhRL-MCT1 and BSG-ChRL interaction. IC50 = 19.5μM.

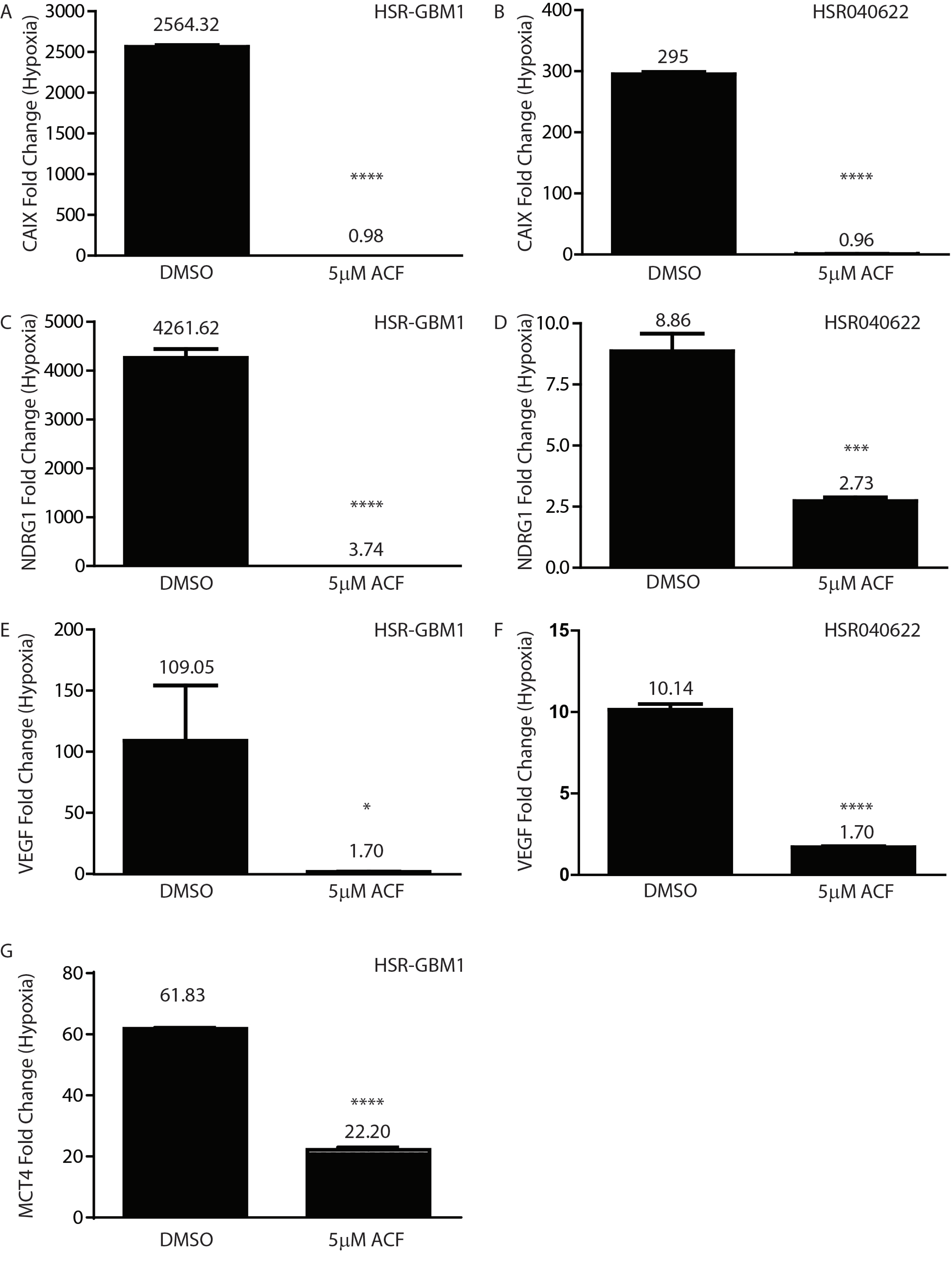

Supplemental Figure S2. ACF inhibits HIF-1 transcriptional activity in GSC lines. Mean +/- SEM is plotted (n=3).
* $P<0.05$; *** $P<0.001$; **** $P<0.0001$ (two-tailed student's t test)

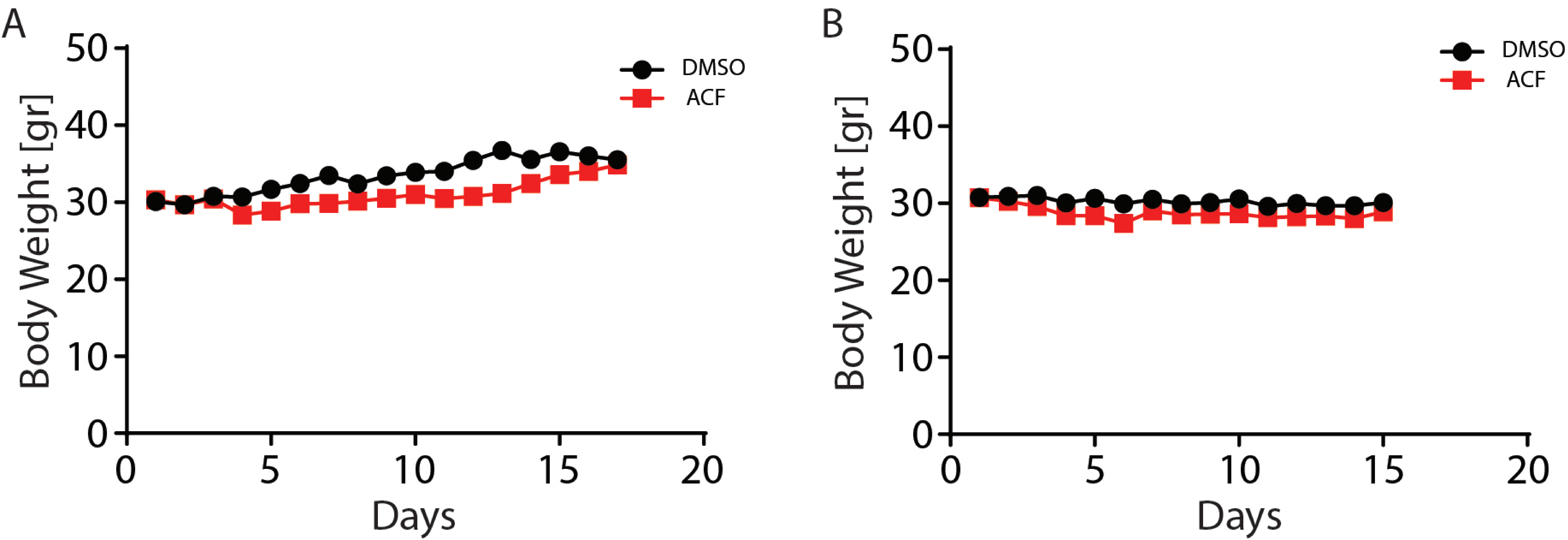

Supplemental Figure S3. Body weights of tumor-bearing mice treated with vehicle or ACF. (A) Weights of tumor-bearing mice from Figure 4A that were treated with vehicle (black) or ACF (red) were measured. Mean + SEM (n = 8 and 7, respectively) is shown. (B) weights of tumor-bearing mice from Figure 5A that were treated with vehicle (black) or ACF (red) were measured. Mean + SEM (n = 11, and 12, respectively) is shown.
